# Supplementary material for: Postoperative avascular necrosis of the femoral head in pediatric femoral neck fractures
Source: PLoS One. 2022 May 12;17(5):e0268058. doi: 10.1371/journal.pone.0268058 (PMC9098045; doi:10.1371/journal.pone.0268058)
Supplement: Attachment — Submitted filename: Response_to_Reviewers.docx [file pone.0268058.s001.docx]

[Date of Resubmission]

Emily Chenette

Deputy Editor-in-Chief

*PLOS ONE*

Dear Wen-Jun Tu,

Thank you for your letter and for the reviewers’ comments concerning our manuscript entitled "**Postoperative avascular necrosis of the femoral head in pediatric femoral neck fractures**" (ID: PONE-D-21-00008).

We have studied the reviewers' comments carefully and have revised our manuscript accordingly, which we hope will meet with approval. Revised texts are marked in red in the manuscript. The main revisions in the manuscript and our responses to the reviewers’ comments are provided below.

Once again, thank you for the letter and for your time.

Sincerely,

Dr. Kelai Wang

Department of Pediatric Surgery, Qilu Hospital of Shandong University

107 Wenhua West Road, Lixia District, Jinan, Shandong 250012, China

Telephone: 86-18560082255

Fax: none

Email: sddxqlyyxewkwkl@163.com

1.

Please ensure that your manuscript meets PLOS ONE's style requirements, including those for file naming. The PLOS ONE style templates can be found at

https://journals.plos.org/plosone/s/file?id=wjVg/PLOSOne_formatting_sample_main_body.pdf and

<https://journals.plos.org/plosone/s/file?id=ba62/PLOSOne_formatting_sample_title_authors_affiliations.pdf>

**Response: Thank you for your comment. We have ensured that our manuscript meets the style requirement of *PLOS ONE*.**

2. We note that you have indicated that data from this study are available upon request. PLOS only allows data to be available upon request if there are legal or ethical restrictions on sharing data publicly. For information on unacceptable data access restrictions, please see http://journals.plos.org/plosone/s/data-availability#loc-unacceptable-data-access-restrictions.

In your revised cover letter, please address the following prompts:

a) If there are ethical or legal restrictions on sharing a de-identified data set, please explain them in detail (e.g., data contain potentially identifying or sensitive patient information) and who has imposed them (e.g., an ethics committee). Please also provide contact information for a data access committee, ethics committee, or other institutional body to which data requests may be sent.

**Response: Thank you for your comment. Because these data contain some sensitive information of patients, such as name, home address, career, and others, they are only stored in the Department of Imaging, Qilu Hospital of Shandong University. These data are available upon reasonable request from the Ethics Committee on Scientific Research of Shandong University Qilu Hospital. If the readers need the data of this study, they should contact the host of this project, Dr. Wang (Email:** [**sddxqlyyxewkwkl@163.com**](mailto:sddxqlyyxewkwkl@163.com）and)**).**

Reviewers' comments:

Reviewer's Responses to Questions

**Comments to the Author**

1. Is the manuscript technically sound, and do the data support the conclusions?

The manuscript must describe a technically sound piece of scientific research with data that supports the conclusions. Experiments must have been conducted rigorously, with appropriate controls, replication, and sample sizes. The conclusions must be drawn appropriately based on the data presented.

Reviewer #1: Partly

**Response: Thank you for your suggestion. We believe that despite less satisfactory statistical efficiency due to the small number of cases, our data analysis of existing cases is rigorous and credible. Therefore, the result of this study is credible.**

2. Has the statistical analysis been performed appropriately and rigorously?

Reviewer #1: Yes

**Response: Thank you for your kind comment.**

3. Have the authors made all data underlying the findings in their manuscript fully available?

The [PLOS Data policy](http://www.plosone.org/static/policies.action#sharing) requires authors to make all data underlying the findings described in their manuscript fully available without restriction, with rare exception (please refer to the Data Availability Statement in the manuscript PDF file). The data should be provided as part of the manuscript or its supporting information, or deposited to a public repository. For example, in addition to summary statistics, the data points behind means, medians and variance measures should be available. If there are restrictions on publicly sharing data—e.g. participant privacy or use of data from a third party—those must be specified.

Reviewer #1: Yes

**Response: Thank you for your kind comments.**

4. Is the manuscript presented in an intelligible fashion and written in standard English?

PLOS ONE does not copyedit accepted manuscripts, so the language in submitted articles must be clear, correct, and unambiguous. Any typographical or grammatical errors should be corrected at revision, so please note any specific errors here.

Reviewer #1: Yes

**Response: Thank you for your kind comment.**

5. Review Comments to the Author

Please use the space provided to explain your answers to the questions above. You may also include additional comments for the author, including concerns about dual publication, research ethics, or publication ethics. (Please upload your review as an attachment if it exceeds 20,000 characters)

Reviewer #1: This paper describes the high risk of developing AVN with more displaced femoral neck fractures (type III and IV) and no incidences in minimally displaced fractures (type I & II). The sample size is quite small compared to other studies that were referenced (28 total with 6 patients having AVN). When looking at reduction quality as an independent factor it would help to determine that in the setting of the degree of displacement as that will likely be directly correlative (ie. less displacement better reduction) therefore this should be included in the analysis - this appears to be addressed by table 3 but it is not clear to the reader. The table formatting is very difficult to follow due to it's embedding within the manuscript - maybe consider changing to a box plot figure demonstrating the correlation coefficient. There are multiple places within the manuscript where words are not spaced apart (ie. page 6 line 112; page 12 line 230). On page 3 line 45 would change this to read "vascular injury to the femoral head is related...

you do reasonably acknowledge the limitations of the study and mention the option of a multicenter study to increase the statistical power.

**Response:**

**Thank you for reviewing our manuscript, ID# PONE-D-21-00008, from which we have benefited both academically and logically. We apologize for the late reply. The major reason for this is that instead of the originally scheduled date in March this year, the submission date of this manuscript was postponed twice due to some reasons. Concerning the issues you pointed and the ones we discovered during revision, our responses are provided below.**

**Once again, thank you for your constructive comments and helpful suggestions.**

**First, in terms of the sample size, the low incidence of this disease and widespread global pandemic have made it difficult to collect related cases, which is one of the reasons we repeatedly postponed submitting this manuscript. Therefore, it is quite challenging to expand the incidence size in the short term.**

**Through an intensive review of related literature, we found that because of the low incidence of this disease, several single-center studies on femoral neck fractures also encountered such difficulty of limited sample size. Some of these articles include:**

**Article 1: Dai ZZ, Zhang ZQ, Ding J, Wu ZK, Yang X, Zhang ZM, et al. Analysis of risk factors for complications after femoral neck fracture in pediatric patients. J Orthop Surg Res. 2020;15(1): 58. doi:** [**10.1186/s13018-020-01587-9**](https://doi.org/10.1186/s13018-020-01587-9)**. PMID: 32075662; PMCID: PMC7029480.**

**The sample size of this article was 44 cases.**

**Article 2: Eamsobhana P, Keawpornsawan K. Nonunion paediatric femoral neck fracture treatment without open reduction. Hip Int. 2016;26(6): 608-611. doi:** [**10.5301/hipint.5000382**](https://doi.org/10.5301/hipint.5000382)**. Epub 2016 May 23. PMID: 27229168.**

**Nine cases were included in this article.**

**Article 3: Bukva B, Abramović D, Vrgoč G, Marinović M, Bakota B, Dučić S, et al. Femoral neck fractures in children and the role of early hip decompression in final outcome. Injury. 2015;46 Suppl 6: S44-47. doi:** [**10.1016/j.injury.2015.10.059**](https://doi.org/10.1016/j.injury.2015.10.059)**. Epub 2015 Nov 17. PMID: 26592094.**

**Twenty-eight cases were included in this article.**

**As the project proceeds, related cases are still being included in this research. Nevertheless, because the follow-up time of some new cases does not meet the inclusion criteria, the sample size in this study has not yet been increased. In the future, more cases are certain to be included to expand the sample size, based on which we will conduct another round of statistical analysis to arrive at more accurate and representative conclusions.**

**Second, concerning the inappropriateness in Form 3, we consulted some professionals in statistics and made careful revisions thereafter. In addition, we further classified the degree of fracture displacement in detail according to the Garden fracture classification: Garden types I and II were regarded as insignificant displacement fractures, Garden type III was regarded as a medium displacement fracture, and Garden type IV was regarded as significant displacement fractures. This led the results of the Fisher exact test and multivariate regression analysis to be more accurate and rigorous. Furthermore, we found that the degree of displacement is an independent risk factor for avascular necrosis of the femoral head, and the quality of reduction is only statistically meaningful in the Fisher exact test, which implies that the main factor for avascular necrosis of the femoral head is fracture displacement and the impact of the reduction quality is relatively small.**

**Accordingly, we have revised the related parts in the manuscript.**

**Meanwhile, we noticed that the 95% confidence interval after binary log analysis is 1.004−70.117. Initially, we thought this high value would affect our conclusions. However, the reference and consultation of similar research literature showed that this may be due to the limited number of cases and the low incidence of such disease. Therefore, such a phenomenon is common in the statistical operations of similar studies:**

**Article 1: (Spence D, DiMauro Jp, Miller PE, Glotzbecker MP, Hedequist DJ, Shore BJ. Osteonecrosis after femoral neck fractures in children and adolescents: analysis of risk factors. J Pediatr Orthop. 2016;36(2): 111-116. doi:** [**10.1097/BPO.0000000000000424**](https://doi.org/10.1097/bpo.0000000000000424)**. PMID: 25730381.)**

**The 95% confidence interval in this article is similar to that of the present study; all approximately 1−70, some even reached 1.08−175.67.**

**Article 2: (Moon ES, Mehlman CT. Risk factors for avascular necrosis after femoral neck fractures in children: 25 Cincinnati cases and meta-analysis of 360 cases. J Orthop Trauma. 2006;20(5): 323-329. doi:** [**10.1097/00005131-200605000-00005**](https://doi.org/10.1097/00005131-200605000-00005)**. PMID: 16766935.)**

**The 95% confidence interval in this article is also relatively large, ranging from 3.53 to 59.85.**

**Article 3: (Li Z, Zhuang Z, Hong Z, Chen L, He W, Wei Q. Avascular necrosis after femoral neck fracture in children and adolescents: poor prognosis and risk factors. Int Orthop. 2021;45(11): 2899-2907. doi:** [**10.1007/s00264-021-05210-2**](https://doi.org/10.1007/s00264-021-05210-2)**. Epub 2021 Sep 21. PMID: 34549321.)**

**The 95% confidence interval in this article is also relatively large, which is 2.31−129.56.**

**Article 4: (Yeranosian M, Horneff JG, Baldwin K, Hosalkar HS. Factors affecting the outcome of fractures of the femoral neck in children and adolescents: a systematic review. Bone Joint J. 2013;95-B[1]: 135-142. doi:** [**10.1302/0301-620X.95B1.30161**](https://doi.org/10.1302/0301-620x.95b1.30161)**. PMID: 23307688.)**

**This article summarizes many previous studies and discovers that the calculated 95% confidence interval values of risk factors have a large range.**

**Therefore, we believe that despite less satisfactory statistical efficiency due to the small number of cases, our data analysis of existing cases is similar to that in the above-mentioned literature, which is also rigorous and credible.**

**Third, we have revised the manuscript accordingly. The revised texts are marked in red. Thank you for carefully reviewing our manuscript.**

**Fourth, we adjusted the structure of the article in line with the general structure of academic papers.**

**The above are my responses to the questions raised by the expert reviewers. We would like to thank the reviewers and editors again for their valuable comments and suggestions, which were valuable in improving the quality of our manuscript.**
