# Supplementary material for: Postoperative avascular necrosis of the femoral head in pediatric femoral neck fractures
Source: PLoS One. 2022 May 12;17(5):e0268058. doi: 10.1371/journal.pone.0268058 (PMC9098045; doi:10.1371/journal.pone.0268058)
Supplement: Attachment — Submitted filename: Response to reviewers.doc [file pone.0268058.s002.doc]

[Date of submission:2022/4/5]

Emily Chenette

Editor-in-Chief

PLOS ONE

Dear Editor

Thank you for your letter and for the reviewer‘s comments concerning our manuscript entitled“Postoperative avascular necrosis of the femoral head in pediatric femoral neck fractures (ID:PONE-D-21-00008R1). Those comments are all valuable and very helpful for revising and improving our paper, as well as the important guiding significance to our researches. We have studied comments carefully and have made correction which we hope meet with approval. Revised portion are marked in red in the paper. The main corrections in the paper and the responds to the reviewer’s comments are as flowing:

Responds to the reviewer‘s comments:

Reviewer #1:

1.Throughout the manuscript there are issues with spacing and punctuation (no space between a word and the parentheses), spaces before commas, etc. This needs to be edited more carefully

Response: Thank you for the suggested. The Manuscript had been edited carefully to move this manuscript closer to publication in the PLOS ONE

2: Abstract: Check spaces throughout the result section. Given that the number of children who has AVN was 6, you cannot state that this study proved correlation between degree of displacement and AVN, you can state the results suggest a correlation and there was significance. The sample size is far to small to claim proof of independent correlation.

Response:  We are grateful for the suggestion. To be more clear and preciseness, We revised the part of Abstract :”Although the development of AVN in children with femoral neck fractures may be related to many factors, the results of this research suggest that there is a significant correlation between displacement degree and AVN occurrence

3: methods: p6 line 103: should state closed reduction and open reduction with or without fixation.

results: p8 line 137-138: eliminate trailing 0s (75% instead of 75.000%), results with non-round numbers should be 2 digits beyond the decimal (7.14% instead of 7.143%). page9 Line 159 should be correlative factors.

Response：Special thanks to you for your good comments. We have re-written this part according to the Reviewer’s suggestion

4: Line 140: "bruising by heavy objects" unlikely to cause a femoral fracture, was it that the object fell on top of the patient? Please clarify this.

Response： I'm sorry for what happened. Because of my confusion, I should have written "crushed by heavy objects "instead of "bruising by heavy objects". It has now been modified. We appreciate for your warm work earnestly, and hope that the correction will meet with approval.

The manuscript has been rechecked and the necessary changes have been made in accordance with the reviewer’s suggestions. Thank you for your consideration. I look forward to hearing from you.

Author’s name: Kelai Wang

Affiliation: Department of Pediatric Surgery, Qilu Hospital of Shandong University, 107 Wenhuaxi Road, Jinan, Shandong, China

Postal address:250012

Phone number: +8618560082255

Email address:sddxqlyyxewkwkl@163.com
